# Moderate Fever Cycles as a Potential Mechanism to Protect the Respiratory System in COVID-19 Patients

**DOI:** 10.3389/fmed.2020.564170

**Published:** 2020-09-11

**Authors:** Anthony Guihur, Mathieu E. Rebeaud, Bruno Fauvet, Satyam Tiwari, Yoram G. Weiss, Pierre Goloubinoff

**Affiliations:** ^1^Department of Plant Molecular Biology, Faculty of Biology and Medicine, University of Lausanne, Lausanne, Switzerland; ^2^Department of Anesthesiology, Hadassah Hebrew University Medical Center, Jerusalem, Israel

**Keywords:** acute respiratory distress syndrome, COVID-19, SARS-CoV-2, fever, Hsp70, heat- shock response

## Abstract

Mortality in COVID-19 patients predominantly results from an acute respiratory distress syndrome (ARDS), in which lungs alveolar cells undergo programmed cell death. Mortality in a sepsis-induced ARDS rat model is reduced by adenovirus over-expression of the HSP70 chaperone. A natural rise of body temperature during mild fever can naturally accumulate high cellular levels of HSP70 that can arrest apoptosis and protect alveolar lung cells from inflammatory damages. However, beyond 1–2 h of fever, no HSP70 is being further produced and a decreased in body temperature required to the restore cell's ability to produce more HSP70 in a subsequent fever cycle. We suggest that antipyretics may be beneficial in COVID-19 patients subsequent to several hours of mild (<38.8°C) advantageous fever, allowing lung cells to accumulate protective HSP70 against damages from the inflammatory response to the virus SARS-CoV-2. With age, the ability to develop fever and accumulate HSP70 decreases. This could be ameliorated, when advisable to do so, by thermotherapies and/or physical training.

## The Effect of Elevated Environmental Temperatures on The COVID-19 Pandemic

In February 2020, many health and political officials across the world were still grossly underestimating the severity of the developing COVID-19 pandemic, in part because of the scientifically unproven belief that like seasonal influenza, COVID-19 would disappear by April 2020 with the rise of temperatures in the Northern Hemisphere. Aside from being scientifically improper to extrapolate information from other seasonal viruses to a novel virus propagating in populations lacking prior immune defenses (1), SARS-CoV-2 was since proven to resist warm summer temperatures in countries of the northern hemisphere, experiencing dramatic deadly second waves of infections (https://coronavirus.jhu.edu/map.html). Moreover, the outbreak of another closely related coronavirus, MERS-CoV, occurred in Saudi Arabia despite scorching spring and summer temperatures (2). Unless strong measures are taken against the COVID-19 pandemic through social distancing and by developing effective vaccines and anti-viral drugs, it is likely to become an ongoing plague. Nonetheless, as previously suggested for SARS-CoV-1 (3), warmer temperatures and higher humidity may reduce SARS-CoV-2's viability *ex vivo* on infective surfaces, thereby mitigating the spread (4, 5). *In vivo* evidence is lacking on mitigating or aggravating effects of high fever on SARS-CoV-2 replication. Yet, cycles of mild harmless (<38.8°C) fever may have strong effects on the disease pathology, as a result of the accumulation of heat-shock proteins (HSPs), in particular of the HSP70 chaperone, which can help the respiratory cells to sustain stress from both the virus and the human inflammatory response to the virus.

## Acute Respiratory Distress Syndrome Is The Major Cause of Death from SARS-COV-2 Infection

By early September 2020, COVID-19 has already caused over 870,000 deaths worldwide. In the most severe cases, the disease progresses into acute respiratory distress syndrome (ARDS), which is among the top three complications after sepsis, causing respiratory failure and death (6). ARDS occurs when protein-rich inflammatory edema fluid builds up in the alveolar space as a result of lung damage, leading to non-cardiogenic pulmonary edema and decreased arterial oxygenation that necessitates mechanical ventilation (7). Early phases of lung pathology in COVID-19 pneumonia show a rather classic edema, with proteinaceous exudates as large protein globules, multinucleated giant cells and hyperplasia of pneumocytes, as with other types of sepsis-induced ARDS. Vascular congestion, combined with inflammatory clusters of fibrinoid material have also been reported, indicating that vascular inflammation and coagulopathy may be more particular hallmarks of the disease (8).

The pulmonary alveoli, which are the main sites of gas exchange with the blood, are composed of a thin alveolar epithelium that covers 99% of the lung surface and includes thin, squamous type I cells (AT1) and cuboid-shaped type 2 cells (AT2). The general hallmark of initial ARDS-induced lung injury is increased capillary leakage and intra-alveolar edema. The AT1 cells that enable gas exchanges undergo irreversible programmed cell death or necrosis, whereas AT2 cells, rather than undergoing limited division and differentiating into new functional AT1 cells, undergo unchecked division and do not differentiate. They accumulate into so-called “ground-glass opacities,” filling the lung cavities and leading to lung failure (9). Treatment of severe ARDS from COVID-19 is an ongoing challenge. Protective mechanical ventilation remains the pillar of ARDS management to facilitate oxygenation with the goal of improving oxygenation through the damaged lungs while reducing ventilator-induced lung injury. If mechanical ventilation fails, extracorporeal membrane oxygenation has been used in COVID-19 ARDS patients with promising results (10).

In the absence of prior effective vaccination (11), another important treatment direction is the prevention or reduction of cell infection by the virus through the repurposing of drugs such as remdesivir, chloroquine, lopinavir/ritonavir, which have different mechanisms of action and are still under development and experimental evaluation (12). Additional therapies aiming at enhancing the natural cellular defenses against the onset of ARDS should be considered. Importantly, mortality from SARS-CoV-2 infections is extremely low among young patients and increasing dramatically in patients aged above 65 (13). For example, official numbers from the end of July 2020 showed that the mortality risk in Switzerland is 150-fold higher for COVID-19 patients aged 70–80+ (1,519 deaths), compared to patients aged 30–49 (10 deaths) [data as from July 28 of the Federal Office of Public Health (FOPH), https://www.bag.admin.ch/bag/en/home.html]. This is evidence that strong natural cellular defenses against the virus are at work in youth which, for reasons yet to be clarified, become progressively less effective in late adulthood possibly in association with genetic parameters, such as gender and blood type (14) and are aggravated by health preconditions, such as obesity, smoking, diabetes and heart diseases.

### The Mitigating Effect of Mild Fever on ARDS

The heat-shock response (HSR) is an example of the buildup of such natural cellular defenses that are highly effective in youth and become progressively less effective in late adulthood. The HSR is defined by the transient accumulation of so-called heat-shock proteins (HSPs), most of which belonging to the conserved chaperone families HSP70, HSP90, HSP60, and HSP40, in response to a temperature rise. HSPs play a general cytoprotective role, among others, in lung inflammation (15). An effective HSR protects thermolabile proteins and membranes from damage caused by excessive variations in the environment, such as heat stress, oxidative stress, UV light, or infection (16, 17). It typically leads to the onset of acquired thermotolerance, i.e., to the transient resistance to a subsequent otherwise deadly dose of elevated temperature (18). It has been shown that, as with externally applied high temperatures, mild fever also activates the HSR in mammals, thereby accelerating healing and preventing apoptosis of respiratory epithelial cells (19, 20). Fever is a major hallmark of inflammatory diseases. Despite its high metabolic cost, it has been an integral part of vertebrate's immune response to infections for the last 400 million years (21), suggesting that fever provides a strong evolutionary advantage for the survival of the fittest. Yet, for over a century, caregivers generally considered fever dangerous and a source of patient discomfort, leading to the systematic use of antipyretics. There is, however, growing evidence that allowing the onset of mild fever leads to better outcomes (19, 22, 23) and higher survival to infectious diseases, especially in cases of ARDS (24, 25).

### High Cellular Concentrations of HSP70s (HSPA1A) Can Repress Inflammation-Induced ARDS

**Figure 1:**
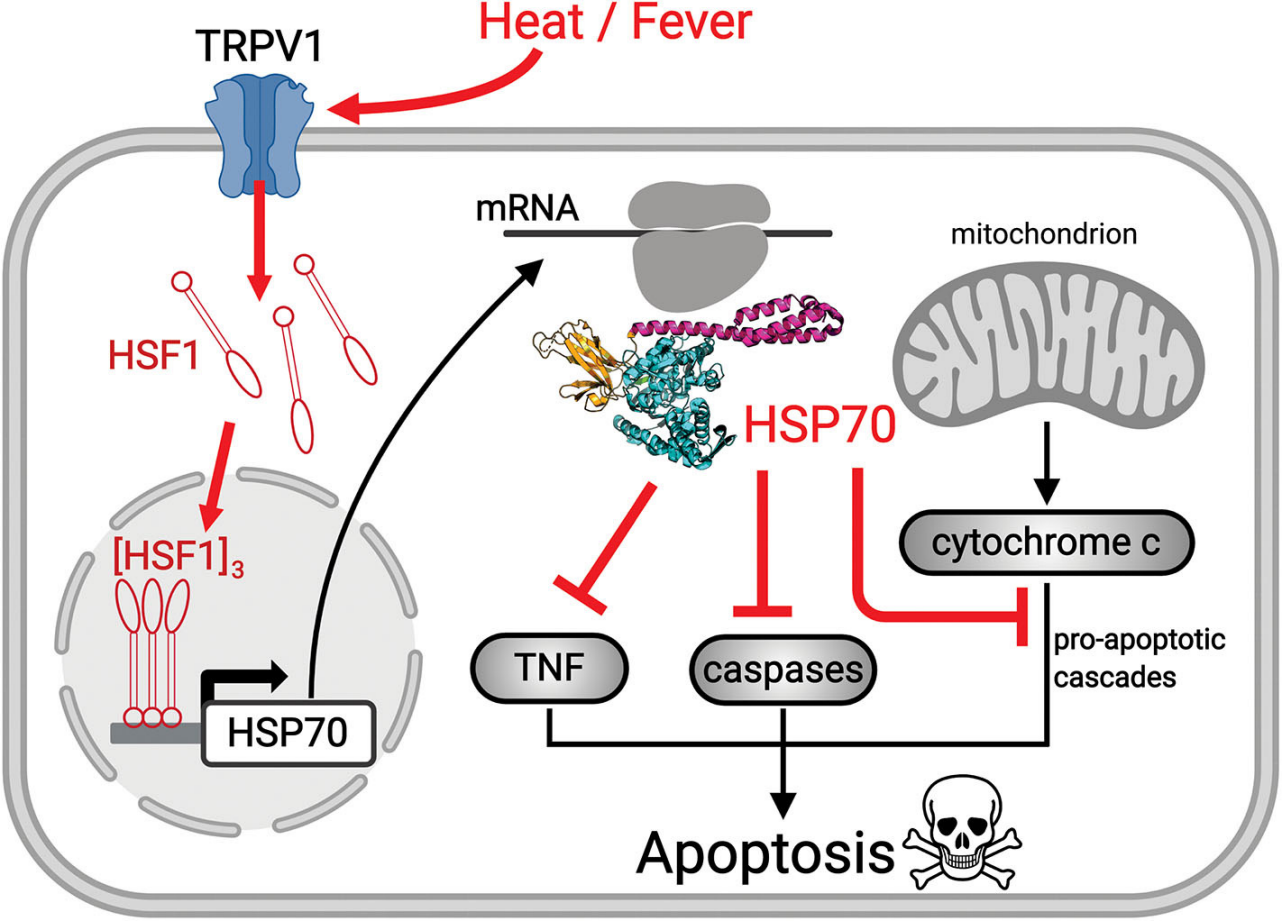
Scheme showing the mechanism of heat-induced HSR and its protective effects. Upon heat-stress such as fever, the fluidized plasma membrane triggers the transient opening of heat sensory calcium channels, such as TRPV1. The ensuing specific calcium-mediated heat-shock signal induces the hyperphosphorylation of cytosolic inactive monomeric HSF1, that trimerizes and translocates to the nucleus where it binds promotor regions of HSP-encoding genes. The mRNA of HSP70 is translated in the cytosol into HSP70 acting to repress sepsis-induced ROS-mediated pro-apoptosis signals (red cross sticks), leading to survival of pre-existing alveolar type 1 cells and to the differentiation of alveolar type 2 cells, into functional, new alveolar type 1 cells.

HSP70s belong to a highly conserved family of molecular chaperones constituting up to 1% of the total protein mass of healthy mammalian cells (26). HSP70s can use the energy from ATP hydrolysis to forcefully unfold and dismantle different types of aggregated and functional protein oligomers in the cell. Hence, it can drive conformational changes in various large cytotoxic protein aggregates and convert them into soluble, harmless, functional proteins (27). Interestingly, HSP70s can also drive the specific dismantling of various active protein oligomers, such as clathrin cages, active heat-shock transcription factor (HSF1) trimers, and active pro-apoptotic IκB oligomers, which become reversibly inactivated upon HSP70-mediated de-oligomerization (28, 29) (Figure 1). Using a rat model for ARDS, it has been shown that an adenoviral vector expressing the stress-inducible form of HSP70, HSPA1A, can effectively protect against sepsis-induced ARDS by limiting neutrophil accumulation in the lungs and causing the inactivation of IκB complexes (30). HSP70 over-expression is also known to efficiently prevent caspase activation, and the heat-induced accumulation of mitochondrial HSP70, HSPA9, also can protect stressed mitochondria (31, 32), thereby conferring cells challenged by pathogens, cytotoxic chemicals, or abiotic stresses, with resistance from ROS-induced mitochondrial- and IκB-associated apoptosis. Hence, cancer cells often resist chemo- and thermo-therapies by over-expressing HSP70 chaperones, HSPA1A in particular (26). Conversely, degenerative neuronal and muscular tissues in aging nematodes and humans that systematically express lower cellular levels of HSP70s than young individuals (33) are particularly fragile and stress-sensitive. Cells with low HSP70 levels tend to spontaneously undergo apoptosis, and consequent tissue losses in aging humans lead to progressive degenerative diseases (34).

**Figure 2:**
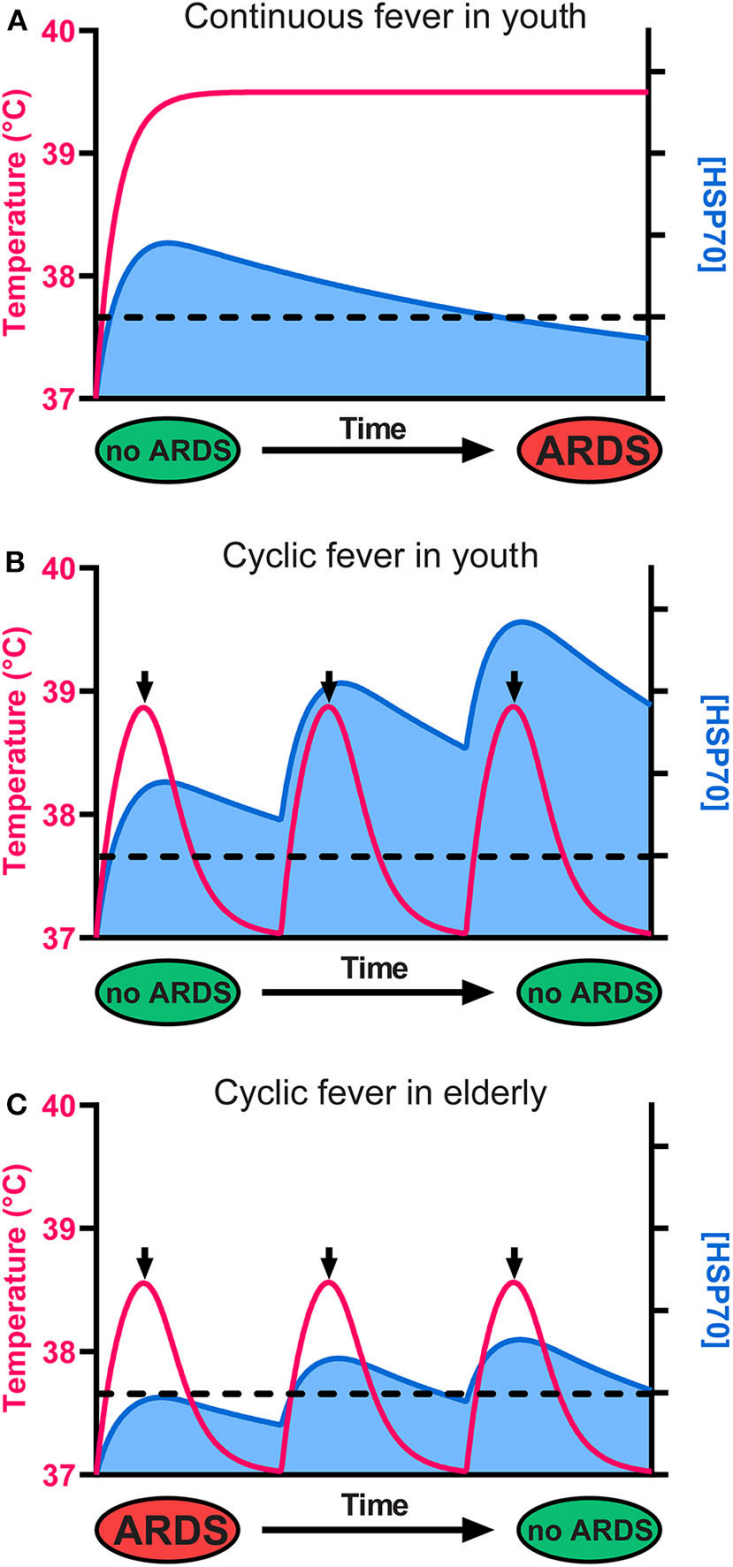
The cellular levels of HSP70s determine the fate of alveolar cells, leading to survival or to ARDS and death. **(A)** Although it initially protects cells from ARDS, a continuous high fever in the long term leads to HSP70 depletion, ARDS, and death. **(B)** In youth, a cyclic mild fever up to 38.8°C (red line) causes in the first hours a strong beneficial accumulation of HSP70s (blue) that can potentially arrest sepsis-induced ARDS in young COVID-19 patients. Because long-term continuous fever leads to HSP70 depletion, the use of antipyretics after 2 h of fever is recommended. Iterative cycles of mild fever can thus maintain and even accumulate high cellular levels of protective HSP70s above a critical threshold (dashed line), preventing ARDS. **(C)** In seniors, the baseline temperature is often lower than 37°C, fever is often less intense, and less protective HSP70s accumulate. However, iterative fever cycles can accumulate protective HSP70s above the critical threshold (dashed line), arresting ARDS even in seniors.

The HSR develops once cells have initially sensed a mild rise in temperature by way of converting small fluidity increments in their plasma membrane, into a specific cellular signal that activates HSF1 and ultimately de-represses HSP-encoding genes, leading to the accumulation of HSPs, the foremost of which being HSPA1A (35, 36) (Figure 1). Noticeably, beyond 2–3 h of continuous heat-shock, cells become ineffective at further accumulating heat-shock proteins and need to stay several hours back at low temperature to reset their ability to effectively respond again to a temperature rise. This implies that under continuous high fever, in the long term, the protective HSP70 molecules will gradually degrade without being replenished and will possibly reach a critically low level that cannot arrest apoptosis (Figure 2A). Interestingly, young COVID-19-infected patients develop ARDS much less frequently than older patients (Figure 2B), mirroring the fact that the HSR and the onset of acquired thermotolerance in humans are optimally effective in youth and progressively fail post puberty, in aging adults (Figure 2C). This can be attributed, in part, to the gradual stiffening of the plasma membranes in aging individuals as a result of decreased physical activity and the excessive intake of highly caloric food containing saturated lipids and cholesterol (37). In addition, the HSR may become impaired with age in particular in neural, liver and muscle tissues (38–42), likely in response to a hormonal signal that initiates at puberty (33, 43).

The combination of insufficiently elevated fever and a less effective HSR in the lung cells of the elderly may thus lead to insufficient cellular amounts of protective HSP70s and the consequent failure to repress apoptosis in ARDS (Figure 2C) (44).

### The HSR Is Transient

**Figure 3:**
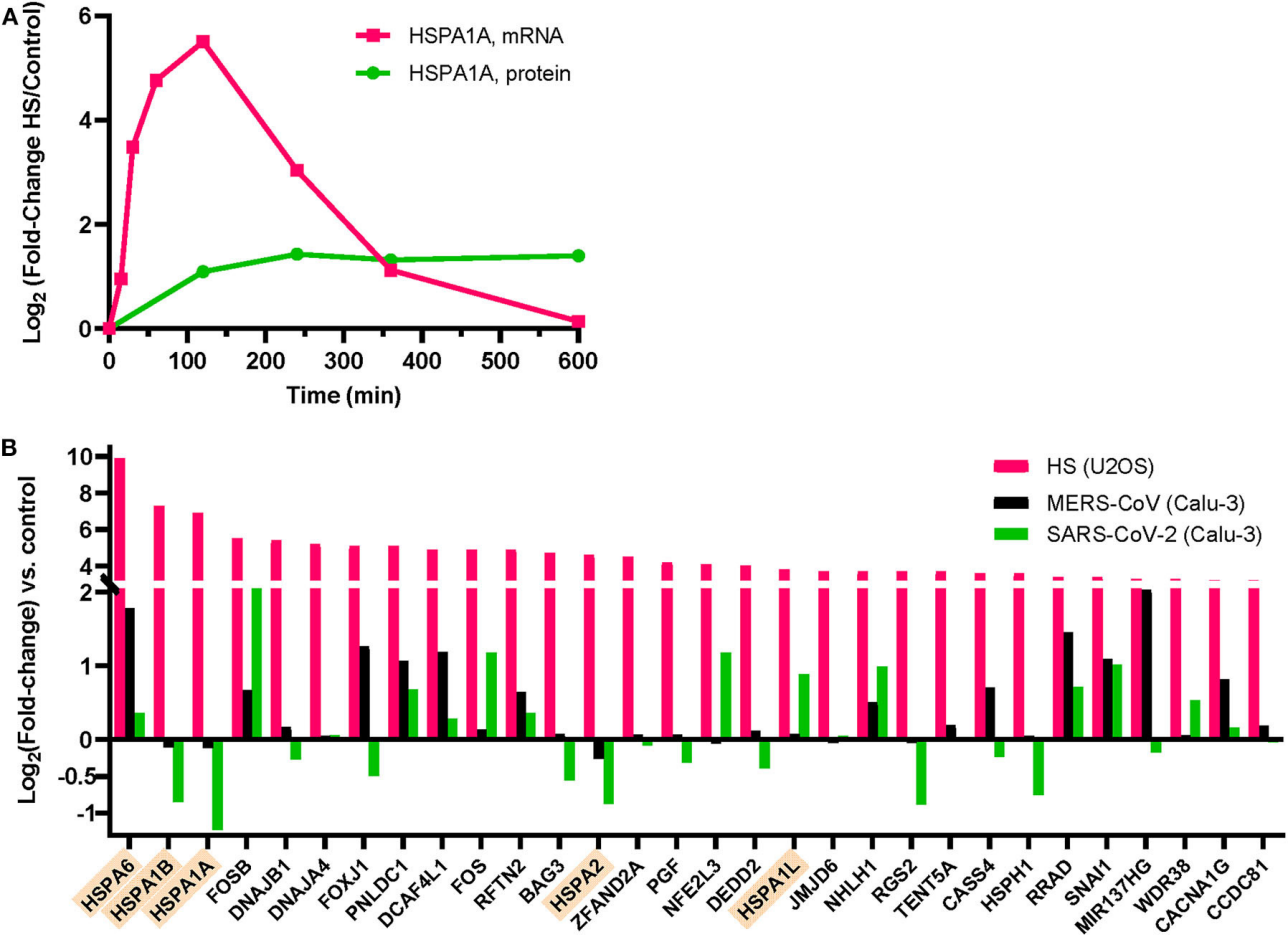
Expression of heat shock proteins (HSPs) in human cells following heat-treatment at 42°C or MERS-CoV infection. **(A)** HSPA1A mRNA (pink) and protein (green) expression in Jurkat cells during a 4 h HS at 41°C, followed by 6 h at 37°C [data from (45)]. HSPA1A mRNA induction is maximal at 2 h HS and thereafter decreases despite the ongoing HS for another 2 h. **(B)** Normalized induction levels of the 30 most heat-induced genes by heat shock (42°C) in U2OS cells (pink) [data from (50)], by MERS-CoV infection (black) of human bronchial epithelial Calu-3 cells (black bars) [data from (51)], or by SARS-CoV-2 infection of Calu-3 cells (green bars) [data from (52)]. Cytosolic HSP70s are indicated in orange.

Whereas, SARS-CoV-2-infected patients experiencing mild fever may optimally accumulate HSP70s in both AT1 and AT2 cells, it should be noted that the HSR is transient. Following the rapid synthesis of HSP70 mRNA within the first ~2 h of a temperature rise and the consequent cellular accumulation of HSPs, mRNA levels start decreasing despite the ongoing elevated temperature (Figure 3A) (45, 46). Hence, HSPA1A mRNA stops accumulating after about 2 h of high fever, and HSPA1A protein levels peak at around 4 h and thereafter start slowly decreasing, despite the ongoing heat shock (Figure 3A) (45). Remarkably, the cells need to return to 37°C for several hours in order for additional HSPA1A to be synthetized in a subsequent fever cycle, to replace the degraded chaperones and thus maintain apoptosis arrest. This behavior results from the fact that the initial step of the heat-shock signaling pathway is the transient opening of heat-sensory calcium channels, called transient receptor potential cation channel subfamily V member type 1 (TRPV1), that become depolarized in response to the heat-induced fluidization of the plasma membrane in which they are embedded (18, 47). Similar to unresponsive pain-depolarized nociceptive channels, and like their heat-sensing plant cognates, the cyclic nucleotide gated channels 2 and 4 (48), the heat-depolarized animal TRPV1 channels need to be returned for several hours at lower temperatures in order to regenerate into fully re-polarized, potent heat-responsive calcium channels (49).

## Extrinsic Heat Treatments and Co-Inducers of The HSR As Potential Prophylactic and Therapeutic Approaches

A mild fever episode of 2–3 h, not exceeding 38.8°C is considered harmless by most of the medical community (53). Moreover, practitioners of traditional medicine on all continents have customarily provided treatments involving controlled mild rising of body temperature, either environmentally applied, as with warm bath therapy (54) that triggers HSP70 accumulation after 1 h at 40°C. Celastrol, a plant triterpene from the Chinese pharmacopeia, has been shown to have cytoprotective effects in autoimmune and inflammatory diseases (55) and could have protective effects against ARDS (56) through HSF1 activation. Defective heat-induced HSP70 production in seniors could be ameliorated prophylactically by increasing their physical training, during which body temperature naturally increases, or by sauna therapy, which can boost the HSR and is reported to reduce the risk for respiratory diseases (57) and for systemic inflammation in this population (58). The protective effects of iterative thermal exercise and increased heat training in marathon runners, in correlation with the induction of heat-shock proteins such as HSP70s and HSP90s, is well-documented (59). During exercise, the accumulation of HSP70s occurs widely across the organism and is measured up to 8-fold in muscle tissues and in lungs (60). However, because heat treatments may be excessively stressful to severely ill SARS-CoV-2 patients, one might expect them to be considered principally as prophylactic, in anticipation of a possible infection. In the context of SARS-CoV-2-induced viral pneumonia, the strategy of increasing cellular HSP70 levels by chemical compounds that could induce or co-induce with mild fever a strong HSR, may be of particular interest, given that both MERS-CoV and SARS-CoV-2 infection was found to specifically and significantly reduce HSPA1A, HSPA1B, and HSPA2 mRNAs in bronchial epithelial cells infected with the virus (Figure 3B) (51, 52).

In the absence of a vaccine at this stage for the COVID-19 pandemic, drug repurposing of FDA-approved molecules could be a timesaver. Several of these, such as carbenoxolone and arimoclomol, have been shown to have beneficial effects in various diseases, mainly by enhancing the heat-induced expression of HSPs (61–63). Glutamine, a conditionally essential amino acid that also triggers the HSR, can improve survival after sepsis and attenuate ARDS symptoms in a mouse model (64).

Were physicians wrong aiming at reducing moderate fever in sepsis? The current knowledge suggests that the answer is yes and no. On the one hand, fever is discomforting to patients and may have adverse effects, especially above 38.8°C. In addition, fever increases oxygen demand, thereby increasing cardiac and respiratory rates (65). A rise of 1°C can increase metabolic demand by 10% (66) and may therefore be detrimental to patients with heart failure or neurological impairment (67). Moreover, antipyretics that are also anti-inflammatory drugs are expected to reduce lung damage caused by an excessive inflammatory response in the lungs, caused by the viral infection. On the other hand, moderate fever, up to 38.8°C, has been reported to inhibit the replication of viruses, such as influenza and other pathogens, promote immunity and most importantly, cause the beneficial accumulation of anti-apoptotic HSP70s that can repress sepsis-induced ARDS. Yet, owing to the transient nature of the heat sensors in the plasma membrane that become depolarized and unresponsive to the heat beyond 2 h of continuous fever, maintaining a fever beyond that time is vain, as the HSP70 molecules that naturally degrade cannot be replenished (Figure 2A). Thus, physicians were not wrong at aiming to reduce fever, as several hours at a low temperature are necessary for the cells to regenerate their heat/fever-depolarized heat sensors and to fully respond again to a new cycle of fever (18, 45). Therefore, based on the above, we hypothesize that the optimal treatment of COVID-19 patients with antipyretic drugs, such as acetaminophen, would be applied only following a couple of hours of moderate fever (Figures 2B,C). Then, several hours at a low temperature would be maintained to allow cells to reset their optimal HSR. Iterative repetitions of such fever cycles, each lasting 8–12 h, may be expected to maintain the highest cellular levels of HSP70s to protect lungs from ARDS damage in COVID-19 patients (Figure 2B) and possibly protect the elderly from ARDS and lung failure (Figure 2C). Prior to any implementation, our hypothesis must be tested in randomized clinical trials with large samples of patients in confinement of similar age and sex with mild symptoms. Antipyretics and their doses should be standardized and the length of the proposed delay before antipyretic intake, allowing mild fever to develop, should be standardized.

One additional avenue of research would be to take advantage of the tissue samples from the nasopharyngeal epithelial mucosa that are routinely used for PCR-based diagnosis of SARS-Cov-2. Quantitative RNAseq of various HSPs, HSP70s in particular, as well as of hallmark genes for ARDS, such as the pro-inflammatory cytokines (IL-1β, IL-6, KC, and MCP-1) (68) and metalloproteinase 9, which is involved in the degradation of extracellular matrix during ARDS (69), and CBIRC3 which inhibits apoptosis (70, 71), may thus be addressed in correlation with the temperature of the patient at the time of sampling, his/her age, gender, the ongoing evolution and final outcome of the disease.

## Conclusion and Suggestions

A large body of scientific evidence now indicates that the accumulation of cellular HSP70s, especially HSPA1A, in lung alveolar cells is beneficial against ARDS-induced lung damage, as in the case of the most severe COVID-19 pathologies. Because mild fever induces the HSR and the accumulation of cellular HSP70s, one would predict that a therapeutic strategy for fever should not be readily decreased by antipyretics. However, fever would optimally need to be thereafter artificially reduced by antipyretics because several hours at 37°C are needed to restore the cellular ability to produce more protective HSP70s in a subsequent fever cycle. Given that age and viral infection may decrease the basal cellular levels of anti-apoptotic HSP70s and further reduce the ability of lung alveolar cells to accumulate HSP70s under stress, we speculate therapeutic strategies should be sought to restore high HSP70 levels in the lung cells of COVID-19 patients. Prophylactic treatments in anticipation of the disease in the elderly could involve natural repetitive stimulations of the HSR in the whole body through controlled intense physical exercise (72–74), sauna therapies and the regular maintenance of calorie-restricted diets (75) containing minimal amounts of saturated lipids and cholesterol.

Interestingly, a prior period of heat acclimation was found in exercising humans to reduce physiological strain and improve physical performance when exercising in moderate normobaric hypoxia (76). Similar effects were shown in rats for which hypobaric hypoxia invoked a cardioprotective heat shock response, consisting of a significant upregulation of HSP70, HSP90, HSP60, and HSP27 that strongly contributed to their survival under acute sub-lethal hypoxia (77). It is tempting to hypothesize that seniors undergoing prior prophylactic treatments of both mild heat-shock and moderate hypoxia, as in daily intense exercising, might increase their ability, once infected, to withstand the increasing hypoxia associated to the acute phases of the disease.

For lack of yet an effective vaccine, the fundamental role in primary care of the COVID-19 crisis is the diagnosis of the suspected COVID-19 patients. In most developed countries this happens via phone calls to detect warning signs, mainly based on the detection of ARDS components and rarely based also on body temperature fluctuations. Given the emerging key role of fever-induced HSP70 expression in the possible mitigation of ARDS damages in SARS-CoV-2 patients, we more pragmatically advocate a systematic research to set precise criteria for temperature monitoring, as a diagnostic feature for initial telemedicine advises and periodic evaluations during self-isolation.

## Author Contributions

AG and BF analyzed published transcriptomic and proteomic data. AG, BF, and PG made the figures. All authors conceived the central ideas of the manuscript, interpreted data from literature, contributed to writing, reviewed, edited, and approved its final version of the manuscript.

## Conflict of Interest

The authors declare that the research was conducted in the absence of any commercial or financial relationships that could be construed as a potential conflict of interest.
